# Bioactivity and antibacterial effect of star anise biosynthesized silver nanoparticles against *Streptococcus mutans*: an in vitro study

**DOI:** 10.1186/s12906-024-04550-x

**Published:** 2024-07-10

**Authors:** Marwa Aly Elchaghaby, Sayed Rashad, Nada Mohamed Wassef

**Affiliations:** 1 Pediatric Dentistry and Dental Public Health, Faculty of Dentistry, Cairo University, Cairo, Egypt; 2 Regional Center for Food and Feed, Agricultural Research Center, Giza, Egypt

**Keywords:** Silver nanoparticles, Anise, *Streptococcus mutans*, Antibacterial, Dental caries

## Abstract

**Background:**

Silver nanoparticles (AgNPs) are receiving a lot of attention as a prospective antibacterial agent for use in caries prevention. The objective of this study was to investigate the bioactivity and antibacterial effect of silver nanoparticles biosynthesized using Star Anise against Streptococcus mutans (S.mutans).

**Methods:**

The bioactive components of the Star Anise were assessed by employing the gas chromatography-mass spectrometry technique. The antibacterial activities of Star Anise Biosynthesized Silver Nanoparticles against S.mutans bacteria were evaluated using Bauer and Kirby’s disc diffusion mechanism and the minimum inhibitory concentration.

**Results:**

Silver nanoparticles biosynthesized using Star Anise revealed high antioxidant activity. AgNPs inhibited S. mutans with a 16 mm inhibition zone diameter and demonstrated an 80 µg/ml minimum inhibitory concentration.

**Conclusions:**

Biologically synthesized AgNPs made from aqueous extract of Star anise appear to be a potential and effective bactericidal agent against S.mutans that can be used to prevent dental caries.

## Introduction

Ayurvedic medicine is one of the oldest therapeutic systems initiated thousands of years ago. Since ancient times, there has been an extended practice of employing plant-based products to improve oral hygiene in various regions. Numerous medicinal plants have been used for years as a traditional cure for various disorders. As a result, efforts to develop substitute medications continue to consider the natural phytochemicals obtained from plants employed in conventional medicine [1].

*Illicium verum* is a Chinese plant from which a cooking spice termed star anise is obtained and is primarily used in Asian countries. Besides its flavor, it has significant medical significance. *Illicium verum* has potent antibacterial capabilities and was once used to treat various illnesses with microbial origins. In addition to having antiviral properties, star anise has gastroprotective, antimicrobial, antifungal, anti-inflammatory, antioxidant, soothing, and spasmolytic effects [2]. Several studies have reported the medicinal uses of star anise, such as proven anticancer effects against breast cancer cells [3], treatment of several diseases of microbial origin [2], dental disease prevention [4], prevention of diabetes-associated complications [5], etc.

The interactions of the various microbial species that make up the oral cavity’s microbiota and oral illnesses are widely known to be related. A reduction in Gram-positive and Gram-negative bacteria is frequently linked to the prevention of dental disease [4].

Globally, the prevalence of oral illnesses, especially untreated dental caries, poses a serious public health issue. Therefore, it is advised to use innovative oral healthcare solutions that can assure sustained improvements in dental health. Utilizing nanomaterial-based technologies for dental therapy is becoming more popular [6].

Herbal medicine has long been used to treat tooth ailments. By addressing the limitations of herbal remedies by synthesizing biogenic metal nanoparticles (MNPs) utilizing plant extracts, the growth of nanoparticle preparations with herbal medicine has become a revolution in dentistry. Ag, Au, and Fe nanoparticles augmented by plant extracts were produced sustainably and outperformed conventional materials in treating various dental conditions [7].

Green synthesized nanomaterials in dental care products have expanded in recent years. Owing to their unique physical and biological characteristics and antibacterial solid activities among other nanomaterials, silver nanoparticles (AgNPs) have drawn interest. Silver compounds have been used for millennia to avert and treat infections, and nanoforms are more effective and biocompatible antibacterial agents. Silver nanoparticles (AgNPs) are widely employed because of their strong ability to kill pathogens and prevent the establishment of microbial resistance [5]. Additionally, AgNPs, due to their larger surface area, can be joined with other treatment agents to display enhanced antimicrobial properties in the oral cavity [6].

The antibacterial activity of silver nanoparticles against several pathogens, including oral pathogen *Streptococcus mutans* [8], Candida *albicans* [9], periodontal pathogens, and strict anaerobes of medical and dental interest [10], *Escherichia fergusonii, Serratia marcescens, and Chromobacterium violaceum* [11], as well as many others, has been previously reported.

*Streptococcus mutans* is a natural resident of the human oral cavity and is one of the leading causes of caries [12]. *S. mutans* is usually regarded as a critical pathogen of human caries and is also associated with periodontal disorders [13].

To the best of our knowledge, there are no available studies regarding the effect of star anise biosynthesized silver nanoparticles on *S. mutans*. Therefore, this study aims to investigate the bioactivity and antibacterial impact of biosynthesized silver nanoparticles against *Streptococcus mutans* using Star Anise.

## Materials and methods

### Plant material and extract preparation

Star anise was obtained in a dry form from the Agricultural Research Center, Egypt. An ultrasonic bath operating at 40,000 Hz was used to sonicate a mixture of 5 g of star anise and 50 ml of deionized water for two hours at 30 °C. After filtering, the extract was kept in the fridge until it was needed again [14]. The extract was subjected to phytochemical screening as described by Afnan et al. [15] HPLC analysis of the extract was carried out using high-performance liquid chromatography (HPLC) Agilent 1260 series.

### Preparation and characterization of AgNPs

A hot plate magnetic stirrer set at 60 °C and 2 ml of star anise extract was combined dropwise with 50 ml of silver nitrate (1 mM) to create the silver nanoparticles solution [16]. An Analytik Jena double-beam UV/Vis spectrophotometer was used to confirm and monitor the synthesis of AgNPs. Zeta Sizer Nano-series (Nano ZS) was used to calculate the particle size of the prepared AgNPs. The prepared AgNPs were also characterized by Transmission Electron microscopy (TEM) and X-ray diffraction (XRD).

### Determination of star anise extract and biosynthesized AgNPs bioactivity

The total antioxidant activity (TAA), total phenolic content (TPC), and total flavonoids (TF) of star anise extract and biosynthesized AgNPs were determined using the phosphomolebdenum method [17], Folin method [18], and aluminium chloride method [19], respectively. Ascorbic acid, gallic acid, and quercetin were employed as reference standards for evaluating TAA, TPC, and TF, respectively.

### Determination of AgNPs antibacterial activity and minimum inhibitory concentration (MIC)

The antibacterial susceptibilities against *S. mutans* ATCC 25,175 bacterial strains were tested in the present study. The microorganisms were acquired from the Microbiology Unit, Microanalytical Center, Cairo University.

The antibacterial activity of AgNPs and Star anise was calculated using Bauer and Kirby’s disc diffusion mechanism. In this experiment, filter paper discs (1 cm) were soaked in AgNP solution. Each bacterium’s 0.1 ml culture suspension, which had been increased to include 10^8^ cells per ml, was seeded into a separate Petri dish after being added to the Muller-Hinton agar medium. One filter paper disc from each treatment was aseptically placed in the center of each plate. At 37 °C, the plates were incubated for 24 h. For each treatment, three duplicate plates were used. Around each disc, the bacterial growth inhibition zones were measured and reported in centimeters [20]. Negative control discs were created using dimethyl sulfoxide (DMSO), while positive control discs used ampicillin as a common antibacterial drug. Each experiment was conducted three times, with the average results being reported.

The MIC for AgNPs against *S. mutans* was determined using a broth-micro dilution method and spectrophotometric assay. The test was done following the Clinical and Laboratory Standards Institute (CLSI) methods. The Clinical and Laboratory Standards Institute (2018) broth technique was used to calculate the MIC of star anise-AgNPs against *S. mutans.* Different concentrations of Star anise-AgNPs were prepared in 0.5% DMSO. Bacterial suspensions (5 × 10^5^ CFU/mL) were added and incubated for 24 h at 37 °C. A blank (sterile culture medium without AgNPs and microorganism suspensions) and a control (sterile culture medium with DMSO) were employed to validate the approach used in this investigation. The minimum inhibitory concentration (MIC) was calculated as the lowest AgNPs concentration that suppressed observable bacterial growth [21].

### Statistical analysis

Statistical analysis was performed by using the mean and standard deviation (SD) using The statistical analysis program Statistical Package for Social Sciences (SPSS) version 22.0.

## Results

### Star anise extract bioactive properties and phytochemicals

Table 1 depicts the antioxidant activity, total phenolic compounds, and total flavonoid content of star anise extract. The results revealed high antioxidant activity and high amounts of phenolic compounds and flavonoids in star anise extract.

**Table 1:** Bioactive properties of star anise extract

TAA (ppm AAE)	TPC (ppm GAE)	TF (ppm QE)
20936.5 ± 54.89	5157.0 ± 20.63	206.5 ± 8.96

TAA: total antioxidant activity; TP: total phenolic compounds; TF: total flavonoids

AAE: ascorbic acid equivalent; GAE: gallic acid equivalent; QE: Querectin equivalent

The different groups of phytochemical compounds identified in star anise extract are given in Table 2. The results confirmed the presence of alkaloids, glycosides, steroids, flavonoids, tannins, saponins, and phenols present in the star anise extracts, whereas glycosides were absent.

**Table 2:** Phytochemical analysis of (Star anise)

Phytochemicals	Star anise extract
Tannins	Present
Flavonoids	Present
Glycosides	Absent
Phenols	Present
Saponins	Present
Alkaloids	Present

The main phenolic and flavonoid compounds determined by HPLC in star anise extract were chlorogenic acid, caffeic acid, rutin, gallic acid and quercetin. The concentrations of these compounds are given in Table 3.

**Table 3:** Phenolic compounds in (Star anise)

Compound	Concentration (ppm)
Gallic acid	235.0
Rutin	405.5
Quercetin	91.55
Chlorogenic acid	183.0
Caffeic acid	26.11

### Formation and characterization of AgNPs

**Fig. 1:**
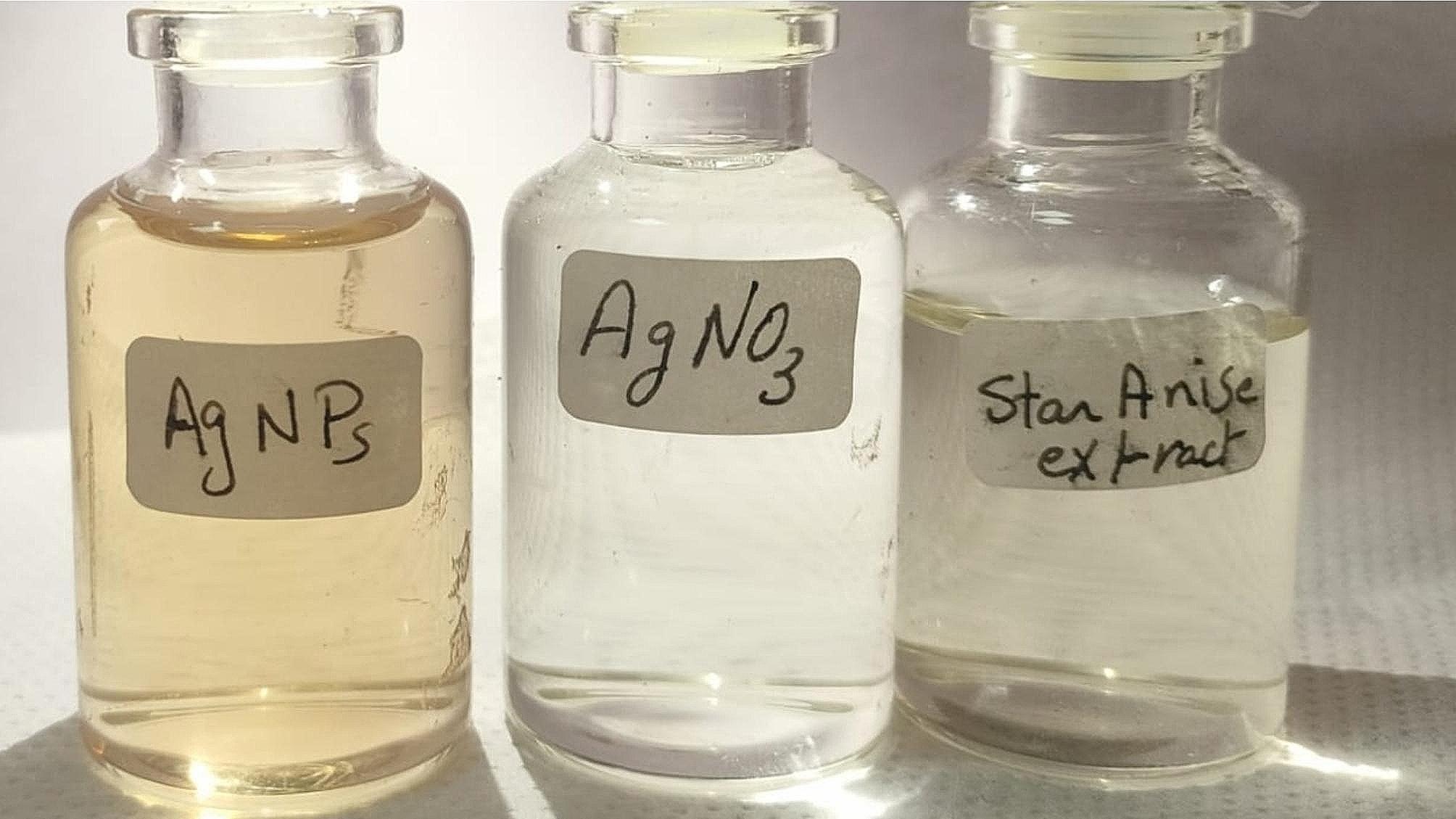
**(a)** AgNO_3_ solution, **(b)** Star anise extract, and **(c)** biosynthesized AgNPs

The formation of AgNPs was visually observed as the color of the solution changed from clear to light yellow-brown (Fig. 1). This color change was interpreted as the first sign of silver photo-reduction and the formation of AgNPs [22, 23].

**Fig. 2:**
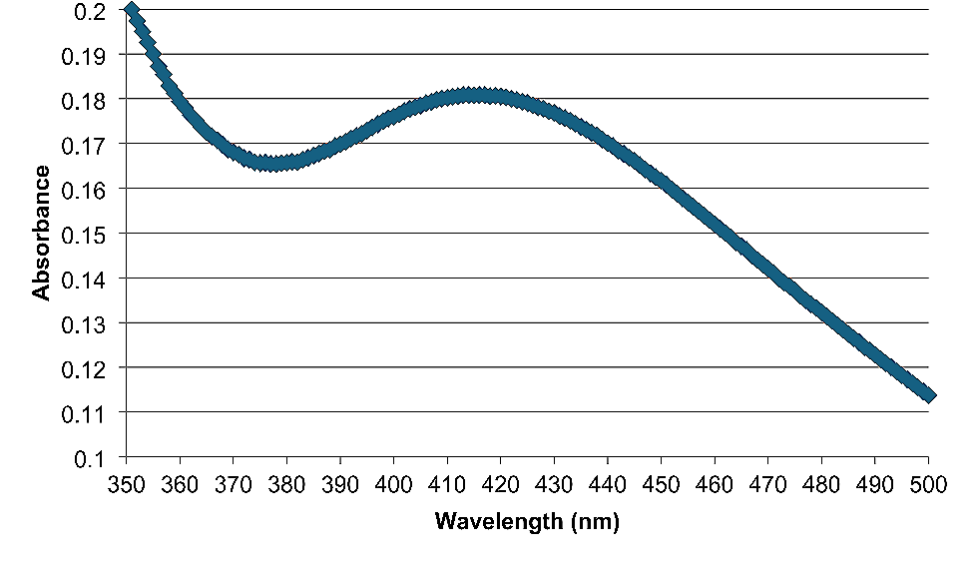
Spectrophotometric spectra of AgNPs after formation

**Fig. 3:**
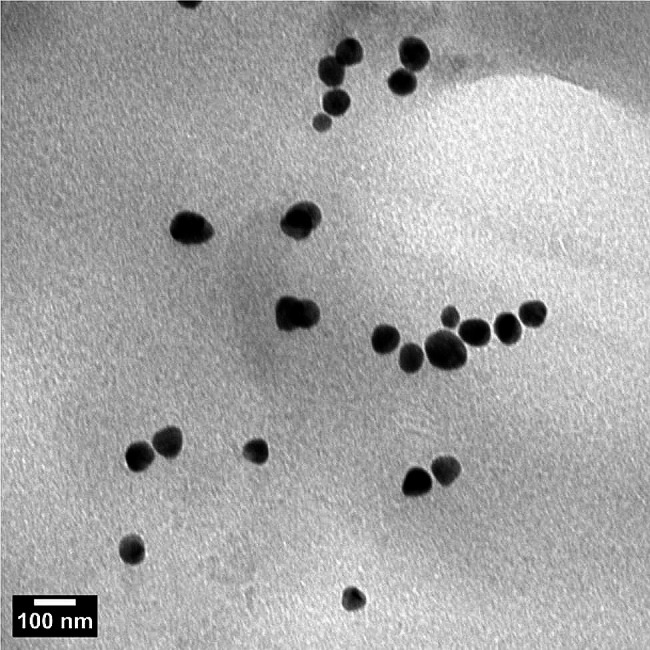
TEM photo of AgNPs

**Fig. 4:**
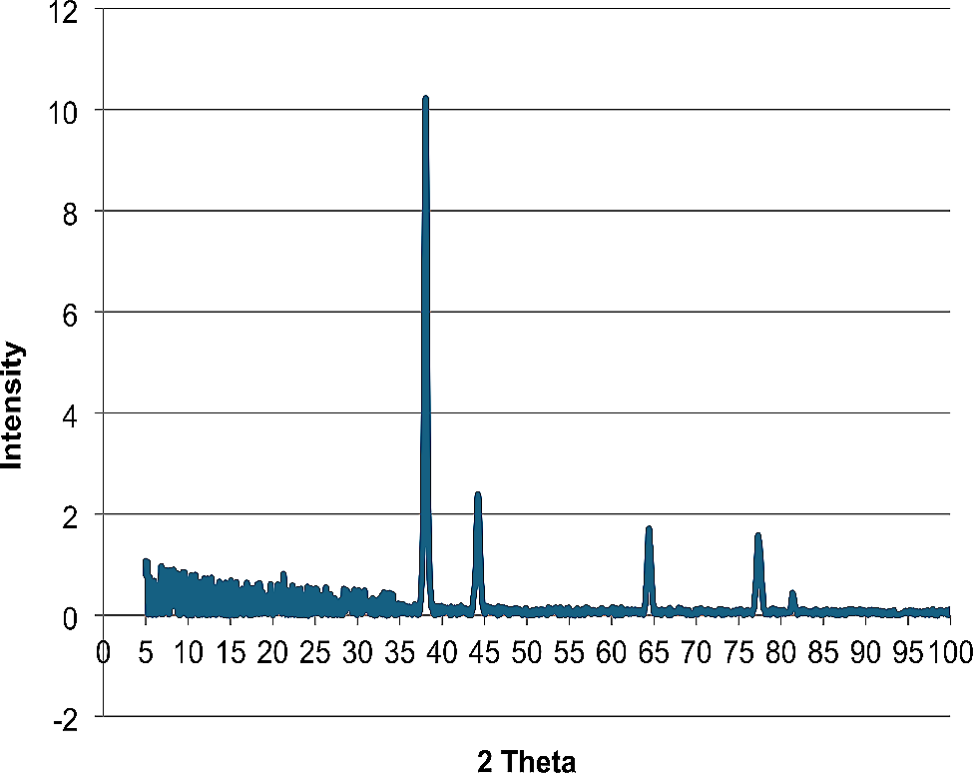
XRD pattern of AgNPs

The UV–visible spectrum of silver nanoparticles biosynthesized using star anise extract (Fig. 2) revealed a maximum peak at 413 nm, corresponding to the plasmon absorbance of silver nanoparticles prepared in the current study. The biosynthesized nanoparticles recorded an average particle size of 66 nm. The formation of AgNPs was further confirmed by transmission electron microscope analysis (Fig. 3) and X-ray diffraction pattern (Fig. 4).

### Antioxidant capacity, total phenolics, and flavonoids content of AgNPs

Table 4 depicts the total antioxidant capacity, phenolics, and flavonoid contents of the biosynthesized AgNPs. The results for the antioxidant activity of the biosynthesized silver nanoparticles revealed that they have good antioxidant activity (23,388 ppmAAE). The results also showed total phenolic compounds (5700 ppmGAE) and total flavonoids (226 ppmQE) comparable to those recorded for star anise extract (Table 4).

**Table 4:** Antioxidant properties of AgNPs

TAA (ppm AAE)	TPC (ppm GAE)	TF (ppm QE)
23388.0 ± 82.33	5700.0 ± 55.98	226.0 ± 4.19

TAA: total antioxidant activity; TP: total phenolic compounds; TF: total flavonoids

AAE: ascorbic acid equivalent; GAE: gallic acid equivalent; QE: Querectin equivalent

### Antibacterial activity of AgNPs against S. mutans

The diameter of the inhibition zone demonstrated that AgNPs biosynthesized with star anise extract showed good antibacterial efficacy against S. mutans bacteria. AgNPs inhibited S. mutans with a 16 mm inhibition zone diameter (Table 5). The minimum inhibitory concentration of AgNPs was found to be 80 µg/ml.

**Table 5:** Antibacterial activity of AgNPs and star anise extract against *S. mutans*

Tested solution	Inhibition zone (mm) (mean ± SD)
DMSO	zero
Ciprofloxacin	38 ± 1.5
AgNPs	16 ± 1.0
Star Anise extract	10 ± 0.5

## Discussion

Dental caries is one of the most public diseases in humans. With the current alteration from the surgical model, which highlighted restorative treatment, to a medical disease management model, novel approaches emphasize disease prevention [24].

*Streptococcus mutans* has been connected as humans’ primary contributory mediator of dental caries. One of its significant virulence properties is the capability to form biofilm identified as dental plaque on tooth surfaces [25]. Consequently, approaches to inhibit numerous factors leading to the virulence properties of *S. mutans* could be an alternative to caries prevention [26].

Several studies evaluating Nanosilver’s antimicrobial effect on *S. mutans* found that Nanosilver had a significant antimicrobial effect on *S. mutans* even at low concentrations [27]. However, the antimicrobial effect of biologically synthesized AgNPs based on star anise extract against S. mutans dental bacteria has yet to be investigated. The current study aimed to biosynthesize AgNPs with star anise extract as a reducing agent and investigate the inhibitory effect of biosynthesized nanoparticles on S. mutans dental bacteria.

Recent studies have demonstrated that green syntheses using biological molecules resulting from plant sources in the form of extracts outclass chemical and/or biological means. To make them appropriate for metal nanoparticle syntheses, these plant-based biological molecules pass over a very controlled assembly process [28]. This study chose Star anise extract as the reducing agent for AgNPs synthesis because it is anti-inflammatory, antibacterial, widely available, inexpensive, easy to obtain, and safe to handle [29].

The cells are shielded from the destructive and damaging effects of reactive oxygen species (ROS) by various antioxidant chemicals that may neutralize free radicals that can cause damage and harm to specific cells. Plants contain free radical scavenging substances such as terpenoids, phenolic compounds, vitamins, and a few additional endogenous metabolites with high antioxidant activity. Plants are rich in flavonoids and phenolic compounds, a primary category of chemicals that operate as principal antioxidants and free radical scavengers [30].

According to the phytochemical study, star anise has high levels of polyphenols and terpenoids, which have diverse biological properties. The phytochemical compounds identified in star anise extract are the main reason for its antioxidant capacity and antimicrobial activity. According to a recent report by Abid and Abachi [31], alkaloids, saponins, and tannins play essential roles in the various antibiotics used to treat common pathogenic strains.

Phenolic acids and flavonoids, commonly known as polyphenolic chemicals and antioxidants, have long been studied, and their discovery rate is increasing due to their positive effects on health and illness [32].

In the present work, several phenolic and flavonoid compounds, such as chlorogenic acid, caffeic acid, rutin, gallic acid, and quercetin, were quantified in star anise extract. This agrees with previously reported data obtained by Iftikhar et al. [33].

According to the current study findings, Star anise has been proven to possess high antioxidant activity and bioactive phenolic and flavonoid compounds that are highly correlated to its antibacterial activity. Also, Anise has antibacterial action by degrading bacterial cell membranes, increasing cytoplasmic permeability, inactivating extracellular enzymes, disrupting electron flow, coagulating cytoplasm, and active transport [34].

Opportunely, using Star anise extract as a reducing agent resulted in biosynthesized AgNPs, which were visually monitored by the formation of brown color in the solution. According to previous research, the appearance of a yellowish-brown color that intensified during incubation indicates the formation of silver nanoparticles. The excitation of surface Plasmon nanoparticle vibrations causes this color change [23, 35].

UV-visible spectroscopy, one of the most important and straightforward methods for confirming nanoparticle formation, was used to verify the biosynthesis of AgNPs. The formation of AgNPs is usually indicated by a maximum spectrophotometric absorbance in the range of 400–460 nm [36]. The size and form of AgNPs are well-known to have a very close relationship with the spectrum of UV-Vis absorption, as previously reported [23]. The presence of a single peak in the spectrum also indicates that spherical nanoparticles are biosynthesized. Furthermore, no other peaks in the spectrum showed that AgNPs were the only formed particles in the solution [37].

Various laboratory methods can be accomplished to assess a pure compound extract in vitro antimicrobial activity. The most famous and basic means are disk diffusion and broth or agar dilution [38]. The disc diffusion method was used in this study because it is a simple and sensitive method that produces categorical results easily understood by all clinicians [39].

The biosynthesized AgNPs revealed good antioxidant potential and its content of phenolic compounds and flavonoids. According to these findings, Star anise is an excellent source of phenolic compounds and flavonoids. The most significant phytochemicals responsible for antioxidant capability are phenolics and flavonoids. The nanoparticles produced utilizing star anise extract demonstrated antioxidant activity due to encapsulated phenolic components [40].

In the current study, AgNPs biosynthesized with star anise extract showed good antibacterial efficacy against S. mutans bacteria. AgNPs inhibited S. mutans with a 16 mm inhibition zone diameter and an 80 µg/ml MIC. AgNPs’ precise antimicrobial mechanism of action has remained unknown until now. However, due to their nanoscale size, AgNPs can easily penetrate microbial cell walls/cell membranes by thiol groups or sulfur-containing proteins, damaging microbial DNA and eventually leading to cell death, according to research [41].

Many researchers have pointed out the application of green or bio-synthesized silver nanoparticles in dentistry. In the current work, AgNPs biosynthesized using Star Anise showed an inhibition zone of 16 mm against S. mutans. In line with this finding, Al-Ansari et al. [8] and Rashad et al. [16] reported an inhibitory action of Synthesised silver nanoparticles using gum Arabic and Spirulina platensis microalgae extract, respectively, on Streptococcus mutans with (18.30 ± 0.5 mm and 12 mm) inhibition zones. Also, de Carvalho Bernardo et al. [10] reported antimicrobial properties of silver nanoparticles and extracts of Syzygium cumini flowers and seeds (13 mm and 7 mm inhibitory zones) on periodontal pathogens. In addition to the previous studies, Al-Fawwaz et al. [42] and Jardón-Romero et al. [43] evaluated the effect of silver nanoparticles from Syzygium aromaticum on some oral pathogens. They showed antimicrobial activity with inhibitory zones of (16 mm and 4 mm) respectively.

By comparing the results of the current study and previous studies found in the literature, it is evident that the natural extract used for the biosynthesis of nanomaterial dramatically influences the resulting bioactivity of this nanomaterial. The interaction of natural compounds contained in plant extracts with nanoparticles results in enhanced antibacterial activity of biosynthesized nanoparticles metallic nanostructures can now be created using natural extracts thanks to the biological synthesis of nanoparticles (NPs), commonly referred to as “green synthesis.” This makes this method of synthesis non-toxic and environmentally benign. On occasion, the properties and cost of NPs produced using this synthesis are on par with or better than those of NPs synthesized using physical and chemical techniques [42].

Based on the results of this study, the biological synthesis of Ag-NPs using an aqueous extract of star anise is a promising and effective antibacterial substance inhibiting S. mutans, one of the main causative factors in promoting dental caries.

## Conclusion

As a result of the above findings, biologically synthesized AgNPs made from an aqueous extract of Star anise appear to be a potential antioxidant and effective bactericidal agent against S.mutans that can be used to prevent dental caries. Further research is necessary to translate this technology into therapeutic and preventive strategies.

## Data Availability

All data generated or analysed during this study are included in this published article.
